# Identification of the Clostridial cellulose synthase and characterization of the cognate glycosyl hydrolase, CcsZ

**DOI:** 10.1371/journal.pone.0242686

**Published:** 2020-12-02

**Authors:** William Scott, Brian Lowrance, Alexander C. Anderson, Joel T. Weadge

**Affiliations:** Department of Biology, Wilfrid Laurier University, Waterloo, ON, Canada; Centre National de la Recherche Scientifique, Aix-Marseille Université, FRANCE

## Abstract

Biofilms are community structures of bacteria enmeshed in a self-produced matrix of exopolysaccharides. The biofilm matrix serves numerous roles, including resilience and persistence, making biofilms a subject of research interest among persistent clinical pathogens of global health importance. Our current understanding of the underlying biochemical pathways responsible for biosynthesis of these exopolysaccharides is largely limited to Gram-negative bacteria. Clostridia are a class of Gram-positive, anaerobic and spore-forming bacteria and include the important human pathogens *Clostridium perfringens*, *Clostridium botulinum* and *Clostridioides difficile*, among numerous others. Several species of Clostridia have been reported to produce a biofilm matrix that contains an acetylated glucan linked to a series of hypothetical genes. Here, we propose a model for the function of these hypothetical genes, which, using homology modelling, we show plausibly encode a synthase complex responsible for polymerization, modification and export of an *O*-acetylated cellulose exopolysaccharide. Specifically, the cellulose synthase is homologous to that of the known exopolysaccharide synthases in Gram-negative bacteria. The remaining proteins represent a mosaic of evolutionary lineages that differ from the described Gram-negative cellulose exopolysaccharide synthases, but their predicted functions satisfy all criteria required for a functional cellulose synthase operon. Accordingly, we named these hypothetical genes *ccsZABHI*, for the Clostridial cellulose synthase (Ccs), in keeping with naming conventions for exopolysaccharide synthase subunits and to distinguish it from the Gram-negative Bcs locus with which it shares only a single one-to-one ortholog. To test our model and assess the identity of the exopolysaccharide, we subcloned the putative glycoside hydrolase encoded by *ccsZ* and solved the X-ray crystal structure of both apo- and product-bound CcsZ, which belongs to glycoside hydrolase family 5 (GH-5). Although not homologous to the Gram-negative cellulose synthase, which instead encodes the structurally distinct BcsZ belonging to GH-8, we show CcsZ displays specificity for cellulosic materials. This specificity of the synthase-associated glycosyl hydrolase validates our proposal that these hypothetical genes are responsible for biosynthesis of a cellulose exopolysaccharide. The data we present here allowed us to propose a model for Clostridial cellulose synthesis and serves as an entry point to an understanding of cellulose biofilm formation among class Clostridia.

## Introduction

Biofilms are communities of microorganisms that reside in an extracellular matrix. This extracellular matrix is produced by the community itself and is composed largely of secreted exopolysaccharides. Biofilms are among the most successful and widely distributed forms of life on Earth, enabling bacteria to adhere to, colonize and persist on a wide variety of surfaces or interfaces. The production of the biofilm extracellular matrix represents a significant resource cost to the organisms producing it, rationalized by the many benefits the biofilm matrix confers. For example, the biofilm matrix serves in resource capture [1,2], accelerated cell growth [3] and the tolerance of stressors or disinfectants, such as shearing forces [2,3], desiccation [2,4], antimicrobial compounds [2,5], extreme temperature [2,5] or sanitizing agents [2,5,6]. Production of an extracellular matrix composed of the exopolysaccharide cellulose has been described in many species of clinical or economic importance, including *Acetobacter* [7], *Clostridium* (previously *Sarcina*) [7,8], *Rhizobium* [7], *Agrobacterium* [7], *Cronobacter* [9], *Salmonella* [10] and *Pseudomonas* [11], among others. The production of a linear β-(1–4)-glucan polymer by these bacteria is accompanied by the derivatization of the polysaccharide into novel biomaterials suited to the needs of the particular organism producing it and the niche being colonized [12]. These exopolysaccharide modifications enhance bacterial persistence and have been identified as a virulence factor under some circumstances [13].

The biosynthesis and export of exopolysaccharides in bacteria is carried out by synthase-dependent systems, encoded on partially conserved operons [14]. A survey of bacterial genomes encoding putative exopolysaccharide synthase operons demonstrated that the evolution of these operon-encoded processes apparently involves multiple gain, loss, duplication, and horizontal transfer events [15]. As a result, these operons are often highly mosaic and frequently involve the acquisition of their components from other biochemical pathways, making their annotation challenging without detailed structural and functional characterization of each synthase component.

At present, an understanding of the synthase responsible for bacterial cellulose biosynthesis has been largely limited to the study of Gram-negative model organisms. The Gram-negative bacterial cellulose synthesis (Bcs) complex is encoded by the *bcsABCZ* locus and represents the essential biosynthetic machinery required for production of the β-(1–4)-glucan polymer [16] that is subsequently derivatized by at least three distinct accessory cellulose modification systems described in Gram-negative bacteria [11,17–19]. The proposed functions of the *bcsABCZ* gene products were largely inferred from structural and functional studies of other Gram-negative exopolysaccharide systems (*e*.*g*., the poly-β-(1–6)-N-acetyl-D-gluccoasmine (PNAG) biosynthetic system from *Escherichia coli* [20] and *Staphylococcal* species [21–23] or the alginate biosynthesis pathway from *Pseudomonas aeruginosa* [24]). Subsequently, structural and functional studies of the Bcs gene products have since provided insight into their functional roles. In these systems, biosynthesis of the glucan polymer is carried out by the family 2 glycosyltransferase (GT-2) BcsA [25]. The processive action of BcsA uses UDP-glucose as a substrate to successively add the sugars to the growing chain, while concomitantly transporting the elongated chain through a pore in BcsA to the periplasm, where it interacts with the carbohydrate-binding module (CBM) contained within BcsB [25,26]. Although the mechanism whereby the nascent cellulose chain is exported from the cell has not been directly elucidated, the BcsC protein is proposed to contain both a β-barrel domain and a tetratricopeptide repeat (TPR) domain to facilitate both the assembly of the Bcs complex and the efficient export of the polymer to the extracellular space [14]. This model is in line with the alginate biosynthesis pathway, where AlgE forms a β-barrel necessary for exopolysaccharide export and AlgK is a TPR-containing protein [27,28]. In the alginate system, release of the polymer from the cell is accomplished by the action of an alginate lyase AlgL [29]. In the Bcs system, this role is accomplished by the family 8 glycoside hydrolase (GH-8) BcsZ [30–32].

Evidence for the production of a cellulose biofilm by the Gram-positive and strictly anaerobic bacterium *Clostridium ventriculi* (formerly *Sarcina ventriculi*) was first reported by Canale-Parola and coworkers [8]. Cultures of *C*. *ventriculi* were observed to form dense microaggregates embedded in an extracellular matrix composed of cellulose and this cellulosic matrix was absent on the surface of planktonic cells not participating in these microaggregates. More recently, a structurally similar biofilm composed of cellular microaggregates was described in *C*. *difficile* 630*Δerm* [33]. Transcriptomic analysis of these microaggregates suggest they are responsive to c-di-GMP because they are associated with the change in expression of a series of diguanylate cyclases and phosphodiesterases, which is a hallmark of the motile-to-sessile transition culminating in biofilm formation. Further, the biofilm-associated transcriptomic changes observed could be partially abrogated by a polar deletion affecting two putative transcriptional regulators. These regulators in *C*. *difficile* share homology with the *Bacillus subtilis* SinR/SinI pair, a known repressor/anti-repressor pair controlling the transcriptional landscape underlying biofilm formation. To date, numerous other Clostridia have been reported to form similar biofilms, including the important human pathogens *Clostridium perfringens* and *Clostridium botulinum*, a phenomenon that has been reviewed by others [34]. Only recently was the Pel polysaccharide biosynthetic gene cluster identified in Gram-positive bacteria, including Clostridia [35]. However, the operon encoding the cellulose exopolysaccharide synthase components has not been identified in any of these species and the specific requirements for biofilm formation in Clostridia remain largely unexplored.

The class Clostridia contains numerous pathogens of global health importance. *C*. *difficile* is one of many such pathogens that is a global threat to public health. *C*. *difficile* infection poses significant morbidity and mortality to all populations worldwide and among individuals beyond the groups traditionally recognized as at-risk (*e*.*g*. the elderly, those under hospital care or those undergoing antimicrobial therapy) [36–38]. The burden of *C*. *difficile* diarrheal disease is also not limited to developing nations; in the United States, *C*. *difficile* caused an estimated 453,000 infections resulting in 29,300 deaths in 2012 alone [37]. Although healthcare-associated costs are challenging to estimate, this burden of *C*. *difficile* infection results in an estimated cost of approximately $6 billion US dollars a year in the United States alone. This data is notwithstanding the other important human pathogens belonging to class Clostridia.

To identify the Clostridial cellulose synthase operon, we surveyed available sequence databases for predicted orthologues of the Gram-negative cellulose synthase components. We identified five hypothetical genes sharing an immediate genomic context that satisfy all known criteria for a cellulose exopolysaccharide synthase, where the cellulose polymerase shares direct homology with the Gram-negative operon, while the remaining subunits represent a mosaic of evolutionary lineages. Furthermore, the composition of this hypothetical gene cluster is conserved within some members of Clostridia. We designate this gene cluster herein as the Clostridial cellulose synthase (*ccsZABHI*) to distinguish it from the Gram-negative Bcs locus. Based on sequence annotation and homology modelling, the locus appears to encode a glycosyltransferase (CcsA), but demonstrates a divergent glycoside hydrolase (CcsZ) and a carbohydrate-binding domain that may aid in polymer translocation (CcsB), with the notable absence of an equivalent to the Gram-negative outer membrane/TPR component (BcsC). Additionally, sequence homology and modelling predicted that the adjacent *ccsH* and *ccsI* may encode a complex for O-acetylation of the cellulose exopolysaccharide, with a distinct evolutionary origin from the otherwise functionally equivalent proteins in Gram-negative bacteria. To investigate if *ccsZABHI* is responsible for cellulose synthesis, we selected the putative glycoside hydrolase (CcsZ) for cloning and expression, as the specificity of this enzyme would provide information about the polymer produced by the operon and for which a structural and functional characterization would be straightforward. The model of the Clostridial cellulose synthesis system we propose here, which was directly corroborated by our studies on CcsZ, represents an entry point to an understanding of cellulose biofilm formation by Gram-positive bacteria and enables future structural and functional studies of other Clostridial cellulose synthesis subunits.

## Materials and methods

### Identification of *ccsZABHI*

Initial identification of the Clostridial cellulose synthase was carried out by searching the NCBI database (https://www.ncbi.nlm.nih.gov) for sequences annotated as BcsA orthologues or by BlastP search (https://blast.ncbi.nlm.nih.gov/Blast.cgi) using the *E*. *coli* K12 BcsA sequence as a query and filtering results for sequences from Clostridia only. We then identified other putative *ccs* ORFs based upon their genomic proximity to BcsA orthologues and through functional annotation, BlastP searches and homology modelling on the PHYRE2 (http://www.sbg.bio.ic.ac.uk/phyre2/) or SWISS-MODEL (https://swissmodel.expasy.org/docs/references) servers [39,40] to ascertain their likely biochemical roles. Sequence alignments and identities were calculated using the BlastP tool. The topology of CcsB was predicted using the TMHHM server, v.2.0 (http://www.cbs.dtu.dk/services/TMHMM/) [41].

### Cloning

The full-length sequence of the hypothetical exopolysaccharide synthesis-associated glycoside hydrolase from *C*. *difficile* (GenPept accession WP_077724661.1), which we designated *ccsZ*, was predicted to have an N-terminal signal sequence according to the PSort server [42] that remains as a single transmembrane anchor in the mature protein. A *ccsZ* plasmid construct lacking the transmembrane region (amino acids 1–29) was synthesized by BioBasic, Inc. (Markham, ON, Canada) in a pET21 vector background so that the expressed soluble portion of the protein (amino acids 30–340) would contain a C-terminal hexahistidine tag (pCcsZ-His_6_).

### CcsZ expression and purification

The expression host, *E*. *coli* BL21-CodonPlus, was transformed with pCcsZ-His_6_ and cells were grown in rich media (32 g tryptone, 20 g yeast extract and 10 g NaCl) containing ampicillin (100 μg mL^-1^) at 37°C with shaking. Expression was induced with IPTG (1 mM) after cultures reached an OD_600_ of at least 0.6 and growth was allowed to continue 16–20 h at 23°C. The cells were collected by centrifugation at 4300 x *g* for 15 min and 4°C. The pellet was resuspended in buffer A (50 mM Tris-HCl pH 7.5, 300 mM NaCl) and cells were lysed with a cell disruptor (Constant Systems) operating at 17 kpsi. Cell lysate was separated by centrifugation at 28000 x *g* for 45 min at 4°C and the supernatant was loaded onto a column containing 2 mL of Ni-NTA agarose (Qiagen) equilibrated with buffer A for 1 h for purification by nickel affinity chromatography. The Ni-NTA column was washed in 50 mL each of buffer A and buffer A with the addition of 20 mM imidazole-HCl, followed by a wash in 50 mL of buffer A with the addition of 50 mM imidazole-HCl. The protein was eluted from nickel affinity chromatography columns using 25 mL of buffer A with the addition of 500 mM imidazole-HCl. Secondary purification was performed by anion exchange chromatography using a HiTrap Q FF 5 mL prepacked column (GE Healthcare) installed on an Akta Start instrument (GE Healthcare). Protein eluted from Ni-NTA resin was dialyzed against anion buffer A (50 mM sodium phosphate pH 7.5) for 12–16 h and passed over the column three times to promote binding. CcsZ-His_6_ was eluted from the column using a gradient of 0–100% anion buffer B (50 mM sodium phosphate pH 7.5, 1 M NaCl). The purity of protein obtained from both nickel affinity chromatography and anion exchange chromatography was routinely assessed using SDS-PAGE. Where needed, purified protein was concentrated in a centrifugal filter unit (Pall Corporation) with a nominal MWCO of 10,000 Da.

### Crystallization and structure determination

Crystallization of CcsZ was carried out at 20°C using the hanging drop vapour diffusion method. Apo-CcsZ crystals were obtained from drops containing 100 mM Tris-HCl pH 8.5, 200 mM calcium acetate and 25% (v/v) poly-ethylene glycol 4000 in a 1:1 ratio with CcsZ protein concentrated to 12.5 mg/mL. Product-bound crystals were obtained from drops containing 100 mM MES pH 6.0, 200 mM calcium acetate and 20% (v/v) poly-ethylene glycol 8000 (PEG 8000) in a 1:1 ratio with CcsZ concentrated to 12.5 mg/mL. Cellotriose was introduced to the drop at a concentration of 5 mM following crystal growth and to cryoprotectants (crystallization buffer supplemented to 30% (v/v) with PEG 8000) just prior to harvesting and vitrification in liquid nitrogen. Data were collected on beamline 08-ID1 at the Canadian Light Source synchrotron. A total of 1250 images of 0.2° Δφ oscillations were collected using incident radiation with a wavelength of 1.0 Å for the apo-CcsZ crystal and a total of 1800 images of 0.2° Δφ oscillations were collected using incident radiation with a wavelength of 1.0 Å for the product-bound crystal. Collected data was processed with XDS [43]. A *P* 2_1_ space group was determined with a single copy of CcsZ in the asymmetric unit using POINTLESS, then scaled using SCALA and data reduction performed using CTRUNCATE [44]. The processed data was solved using the molecular replacement technique with the Phaser tool in PHENIX [45] using *Tm*Cel5A (PDB ID 3AMD) as a search model. Structural refinement was performed using iterative rounds of automated refinement using the Refine tool in PHENIX, followed by manual refinement and checking of waters using Coot [46]. Refinement progress was monitored by the reduction and convergence of *R*_work_ and *R*_free_. Ligand fitting was performed by LigandFit in PHENIX, followed by calculation of an omit map by Polder in PHENIX. Ligand real-space refinement was performed in Coot using the Polder map and validation was performed using MolProbity in PHENIX. Structure interface analysis was performed using the PDBePISA server (https://www.ebi.ac.uk/pdbe/pisa/pistart.html) [47]. Figures were prepared with PyMOL.

### Glycoside hydrolase activity assays

Activity was initially assessed qualitatively by a plate-based zymogram analysis. Zymogram matrices in agar plates were prepared by heating a solution of 1.5% (w/v) agar with individual carbohydrates (0.8% (w/v) for CMC, xylan and hydroxyethylcellulose; 0.4% for curdlan) to 100°C for 10 min. Spotted on the agar plates were 0.5 mg each of purified CcsZ, cellulase cocktail from *Trichoderma reesei* (Sigma; C8546) and bovine serum albumin (BSA; BioShop). BSA served as the negative control and the *T*. *reesei* cellulase cocktail served as the positive control. The zymograms were incubated either 1 h or 24 h, as specified, at 37°C. Following incubation, plates were stained in 5 mL of a 0.1% (w/v) solution of Congo Red for 1 h and destained in three 5 mL volumes of a 1 M solution of NaCl for 1 h total.

The activity of CcsZ was quantitatively monitored using the dinitrosalicylic acid (DNS) method. Substrates tested included CMC, xylan, arabinoxylan, β-glucan, lichenin, xylan and curdlan. All carbohydrates not purchased from Sigma as noted above were supplied by Megazyme. All substrates were tested at 8.3 g/L, with the exception of curdlan that was tested at 0.75 g/L due to solubility constraints. CcsZ (20 μM) was mixed with substrate in assay buffer (50 mM sodium acetate pH 4.5) for 30 min at 37°C. Reactions were stopped with the addition of 56.3 μL of DNS reagent (1% w/v dinitrosalicylic acid, 4 mM sodium sulfite, 250 mM NaOH) and were heated at 90°C for 15 min. A volume of 18.75 μL of (40% w/v) sodium potassium tartrate was added to stabilize the DNS reaction. The resulting solutions were measured for absorbance at 575 nm in a Cytation5 imaging microplate reader (BioTek). Absorbance values were related to millimolar reducing sugar equivalents using a standard curve of D-glucose (BioShop) measured using the same DNS method. Buffers used for the pH assay included sodium acetate (pH 4.0–5.5), MES (pH 5.5–7), sodium phosphate (pH 7.0–8.0), Tris-HCl (pH 8.0–9.0) and CHES (pH 9.0–10) in increments of 0.5 pH units with each buffer at 50 mM final concentration.

The *endo*- vs *exo*- preference of CcsZ was evaluated using the Megazyme Cellulase Assay Kit (K-CELLG5-4V). Thermostable *exo*-β-glucosidase (provided by the manufacturer) was added to a solution containing 4,6-(3-ketobutylidene)-4-nitrophenyl-β-D-cellopentaoside in 10% (v/v) dimethylsulfoxide and 0.02% (w/v) sodium azide. A 100 μL amount of this solution was pre-incubated at 40°C then inoculated with 100 μL of CcsZ solution (prewarmed to 40°C and containing 15 μM enzyme) in acetate buffer (100 mM, pH 4.5) containing 1 mg/mL bovine serum albumin. This process was performed in parallel with the cellulase cocktail from *Trichoderma reesei* as a positive control and CcsZ without *exo*-β-glucosidase as a negative control. All reactions were incubated at 40°C for 10 min and then 3 mL of stopping reagent (2% (w/v) Tris buffer, pH 10) was added. A volume of 100 μL of the solution was then transferred to a 96-well plate where the absorbance was recorded at 400 nm using a Citation 5 Imaging Reader (BioTek). All model fitting and statistical analyses were performed using GraphPad Prism v8.4.3.

### LC-MS

Liquid chromatography–mass spectrometry analyses were performed at the Mass Spectrometry Facility of the Advanced Analysis Centre, University of Guelph on an Agilent 1200 HPLC liquid chromatograph interfaced with an Agilent UHD 6530 Q-TOF mass spectrometer. A C18 column (Agilent Extend-C18 50 mm x 2.1 mm 1.8 μm) was used for chromatographic separation. The following solvents were used for separation: water with 0.1% (v/v) formic acid (A) and acetonitrile with 0.1% (v/v) formic acid (B). The initial mobile phase conditions were 10% B, hold for 1 min and then increase to 100% B in 29 min. These steps were followed by a column wash at 100% B for 5 min and a 20 min re-equilibration. The mass spectrometer electrospray capillary voltage was maintained at 4.0 kV and the drying gas temperature at 250°C with a flow rate of 8 L/min. Nebulizer pressure was 30 psi and the fragmentor was set to 160. Nozzle, skimmer and octapole RF voltages were set at 1000 V, 65 V and 750 V, respectively. Nitrogen (> 99%) was used as the nebulizing, drying and collision gas. The mass-to-charge ratio was scanned across the m/z range of 50–3000 m/z in 4 GHz (extended dynamic range) positive and negative ion modes. Data were collected by data independent MS/MS acquisition with an MS and MS/MS scan rate of 1.41 spectra/sec. The acquisition rate was set at 2 spectra/s. The mass axis was calibrated using the Agilent tuning mix HP0321 (Agilent Technologies) prepared in acetonitrile. Mass spectrometer control, data acquisition and data analysis were performed with MassHunter® Workstation software (B.04.00).

## Results and discussion

### Identification of the Clostridial cellulose synthase

**Fig 1:**
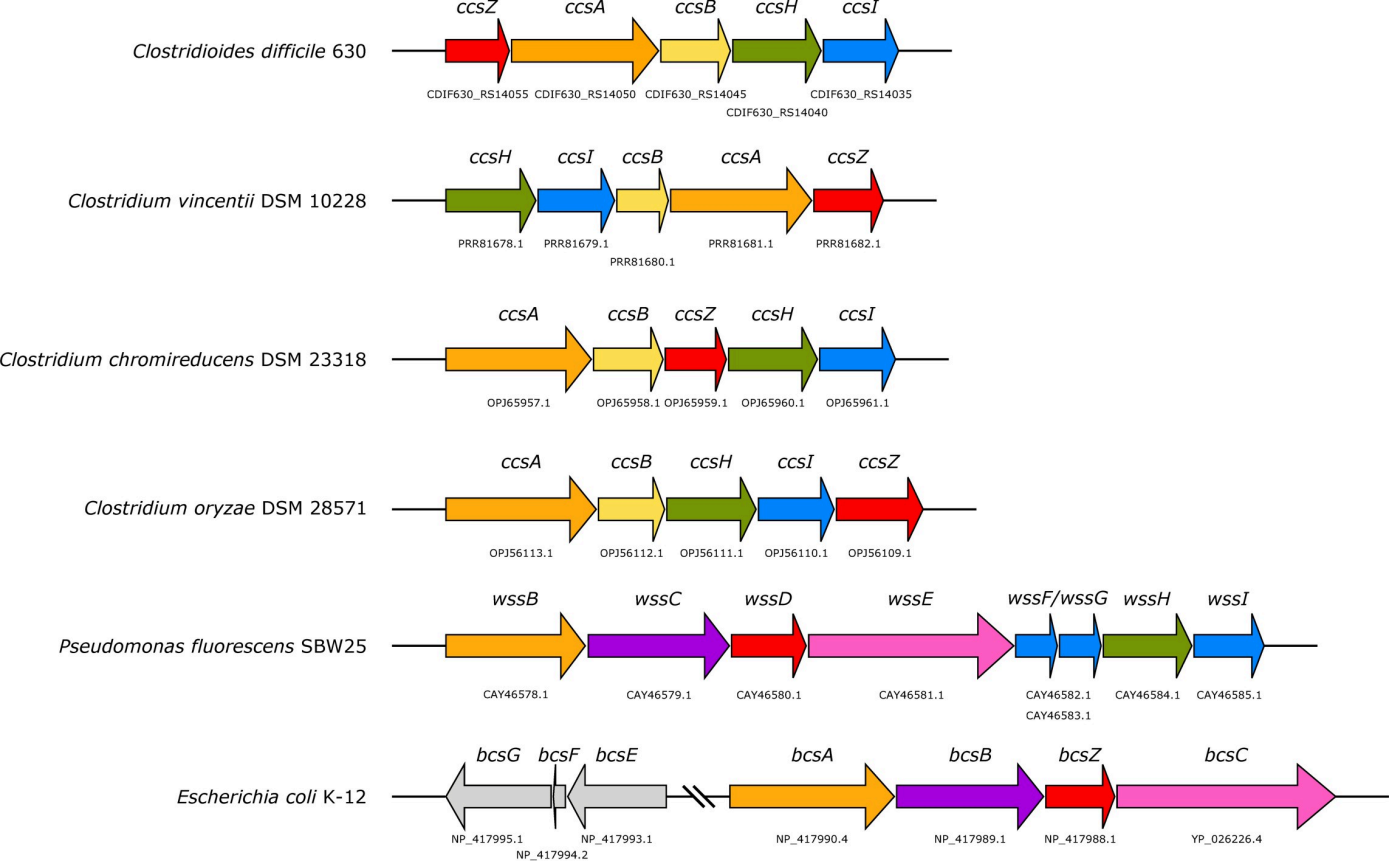
The conserved *ccsZABHI* locus. A set of 5 genes proposed to be involved in *O*-acetylated cellulose exopolysaccharide biosynthesis was identified in *Clostridioides difficile* 630 and appears conserved in other bacteria of class Clostridia. These genes are not homologous to the described Gram-negative cellulose synthase components, but instead comprise a mosaic of distinct evolutionary lineages that encode apparent functional equivalents, including equivalents to the glycosyltransferase BcsA (orange) and the glycosyl hydrolase BcsZ (red), in addition to a putative MBOAT (green) and an O-acetyltransferase (blue) that may O-acetylate cellulose exopolysaccharides, as in *P*. *fluorescens* SBW25. The accession codes (GenBank) for genes are listed below each gene.

We searched the NCBI Protein database for hypothetical proteins that were functionally annotated as cellulose synthases, or which showed homology to known Gram-negative BcsA proteins among the class Clostridia. We identified sequences functionally annotated as putative cellulose synthase catalytic subunits in *C*. *difficile* (GenBank CDIF630_RS14050), *Clostridium vincentii* (PRR81681.1), *Clostridium chromireducens* (OPJ65957.1) and *Clostridium oryzae* (OPJ56113.1), among others (Fig 1; orange). The functional annotations are based on sequence similarity to the GT-2 domain of *E*. *coli* BcsA (UniProt P37653) and the presence of a predicted PilZ domain for binding c-di-GMP, which is a hallmark of synthase-dependent exopolysaccharide catalytic subunits. These features were a promising indication that these proteins were orthologues of BcsA. Homology modelling of these hypothetical proteins also suggested they belong to GT-2, with the structure of BcsA from *Rhodobacter sphaeroides* (PDB ID 4HG6) selected as the top scoring homologue and the template for homology modelling in all cases. To distinguish this locus from the Gram-negative cellulose synthase, we named it the Clostridial cellulose synthase subunit A, or *ccsA*. However, homologs of the remaining Bcs proteins from Gram-negative bacteria (BcsB, BcsC, and BcsZ) were notably absent. Instead, we assessed the genomic context of *ccsA* to identify hypothetical genes that may be functionally equivalent to these proteins, but could have arisen from divergent evolution of the Gram-positive cellulose synthase operon. Exopolysaccharide synthase operon evolution has been shown to involve multiple loss, duplication, rearrangement, and horizontal transfer events, resulting in highly mosaic operon classes distributed among bacterial phyla. Accordingly, we did not find it surprising that the Clostridial cellulose synthase shared no apparent evolutionary relationship with the known Gram-negative operons given the significant phylogenetic distance between the species in question [15].

A survey of the genomic context of *ccsA* sequences demonstrated a series of four other hypothetical genes that always occurred with *ccsA* (Fig 1). These genes included those that are annotated as a glycosyl hydrolase (endocellulase) (Fig 1; red), a gene of unknown function (Fig 1; yellow), a membrane-bound O-acyltransferase family protein (Fig 1; green), and a DHHW family protein (Fig 1; blue). Each of these discrete genes we identified in the conserved *ccs* locus represented pairwise reciprocal best-hits of each other in the other respective genomes we identified them within, suggesting co-evolution of these genes as functionally co-operative operon subunits among the Clostridia.

Homology modelling of the predicted glycosyl hydrolases suggests they belong to the CAZy family glycosyl hydrolase family 5 (GH5) with a variety of enzymes from this family occurring as the top scoring alignments and modelling templates. Glycosyl hydrolases are highly conserved components of exopolysaccharide synthase operons and are thought to be required for release of the polysaccharides into the extracellular space, as well as possibly playing a role in release or dispersion from the biofilm matrix [14,31,48]. We named this hypothetical protein CcsZ, to distinguish it from its functional equivalent in the Gram-negative cellulose synthase operon, BcsZ, which is itself a member of glycosyl hydrolase family 8 [30].

**Fig 2:**
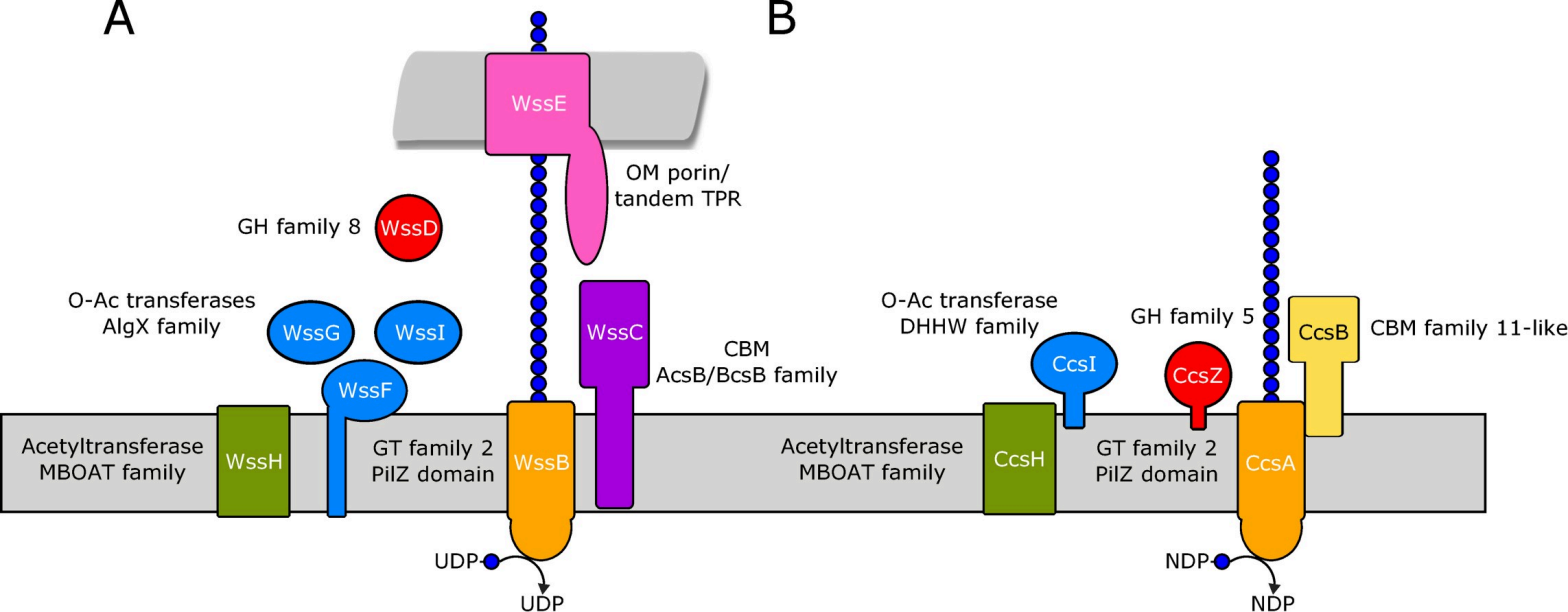
The *ccsZABHI* gene cluster shares predicted structural and functional similarity, but not homology, to the Gram-negative *O*-acetylated cellulose synthase. Proteins are coloured as in Fig 1. (A) The model of the *P*. *fluorescens* SBW25 cellulose synthase based on Spiers *et al*. [11] that contains putative *O*-acetyltransferases resembling those of the alginate biosynthesis pathway in *P*. *aeruginosa*. (B) Our proposed model of the cellulose synthase from selected Clostridia. The synthases we identified (CcsA) contain the hallmark GT-2 and PilZ domains of Gram-negative cellulose synthases, in addition to CcsB, a membrane-bound extracellular protein of unknown function. O-Acetylation of cellulose in this pathway is likely carried out by CcsH and CcsI, which resemble PatA1 and PatB1 from *B*. *cereus*, as well as WssH and WssI from *P*. *fluorescens* SBW25, and cleavage is carried out by the GH-5 enzyme CcsZ reported here.

BLAST searches and homology modelling of the protein of unknown function encoded adjacent to *ccsA* returned no known homologs outside of Clostridia. Homology modelling of this sequence suggested the N-terminal region aligns to structurally resolved family 11 carbohydrate binding module (CBM11) domains and probably adopts a similar β-jelly roll fold. These CBM11 proteins are found exclusively among Gram-positive bacteria and are known to bind mixed linkage β-glucans with high specificity [49]. We named this gene *ccsB* because it is not homologous to the Gram-negative BcsB, but its predicted three-dimensional fold and putative function align with that of BcsB in aiding the translocation of the nascent exopolysaccharide across the cytoplasmic membrane [25,50]. Our predictive model would then imply that CcsB (Fig 2; yellow) would be localized to the cytoplasmic membrane and would form a stable complex with CcsA that is both necessary and sufficient for cellulose synthesis (as observed for BcsA/BcsB). To predict the topology of CcsB, we analyzed its sequence with the TMHMM bioinformatics tool, and found CcsB contains two predicted transmembrane helices of 19 and 22 residues in length. Each of these predicted transmembrane helices reside near the termini of the protein; thereby bracketing the region that aligned to known CBM11 domains. In addition, the region between these helices with the putative CBM domain is predicted to be extracellular, which further positions CcsB as functionally and topologically analogous to BcsB.

We also identified two additional genes that were always encoded in the immediate genomic context of *ccsA*, *ccsB* and *ccsZ*. BLAST and homology modelling analysis of these two genes provided strong evidence that the hypothetical proteins encoded by these genes belong to the membrane-bound O-acyltransferase (MBOAT) family and the DHHW family (Figs 1 and 2; green and blue, respectively). The former is a family of enzymes found in all domains of life, which are responsible for cell surface modification in bacteria [51] and in some vertebrates are responsible for lipid biosynthesis and/or remodelling [52,53]. The latter DHHW family are found exclusively in bacteria and have demonstrated roles in the O-acetylation of cell surface polysaccharides [54]. Taken together, these findings suggest a role for these proteins in O-acetylation of the cellulosic material produced by the adjacent hypothetical operon subunits. The enzymatic modification of many exopolysaccharides have been described and have been reviewed expertly by others [12]. Among those operons that produce modified exopolysaccharides are at least three Gram-negative cellulose operons, which produce crystalline cellulose [18,55], phosphoethanolamine cellulose [17,19,56] and O-acetylated cellulose [11,57]. In Gram-negative bacteria, the operon subunits of *Pseudomonas fluorescens* responsible for O-acetylation of cellulose exopolysaccharides (Fig 1) are homologous to those involved in O-acetylation of alginate [11], a distinct exopolysaccharide found in *Pseudomonas aeruginosa* [58,59]. Bundalovic-Torma and colleagues have proposed that the acetylated cellulose operon class emerged because of the genomic co-occurrence of alginate operons among Pseudomonads, thereby resulting in subsequent duplication and operonic acquisition of the subunits required for O-acetylation [57]. Since no alginate operons have been identified in Clostridia from which these operon subunits could be acquired, this evolution by duplication and operonic acquisition of alginate machinery would account for the lack of homology between Gram-negative acetylcellulose operons and the MBOAT/DHHW pair we identified as part of the *ccs* locus. Instead, we propose that these putative O-acetyltransferase proteins evolved by duplication and operonic acquisition of secondary cell wall modifying complexes that are known to involve the functional contribution of MBOAT/DHHW family proteins in tandem [54]. In keeping with the naming of operon subunits based on their functional equivalence, rather than their evolutionary relationships to the Gram-negative cellulose operon, we named the MBOAT/DHHW pair CcsH and CcsI, respectively, as our model predicts them to be functionally equivalent to WssH and WssI (Figs 1 and 2) [11].

Surprisingly, the systematic pipeline for classifying bacterial operons reported by Bundalovic-Torma and colleagues did not identify the *ccs* operon we describe here. This was a direct result of considering only those loci that contained both a polysaccharide synthase subunit and one additional locus involved in exopolysaccharide modification or transport as defined previously [15]. As our findings above suggest, the additional *ccs* operon components are not homologous to their Gram-negative functional equivalents and no Gram-positive operon subunits were used in the search because none had been identified *a priori* to that study. Instead, Bundalovic-Torma and coworkers identified a unique putative cellulose “operon” in *C*. *difficile* 630 composed of only homologs of BcsA and BcsB, which shared genomic context. To investigate the possible existence of multiple cellulose operons in *C*. *difficile* 630, we assessed the genomic context of this BcsA/B pair proposed by Bundalovic-Toma and coworkers using our multiple-homology approach. We identified that this putative cellulose synthase BcsA from *C*. *difficile* 630 in fact aligns most closely with a related family 2 mannosyltransferase, although it does appear to share more distant homology with BcsA as well. Further, in the genomic context of this putative BcsA/B pair, we identified a putative glycosyl hydrolase belonging to family GH113, a WecB UDP-GlcNAc 2-epimerase homolog and a predicted polysaccharide deacetylase with distant homology to the carbohydrate esterase family 4. Given that i) family GH113 is exclusively composed of endo-mannanases, ii) the polysaccharide polymerase is most closely related to a mannosyltransferase and iii) that a UDP-GlcNAc 2-epimerase is required to produce UDP-ManNAc as an enzymatic precursor to polymer synthesis, these findings suggest that this predicted operon in fact encodes a complex for the polymerization, export, and modification of a partially deacetylated ManNAc-rich exopolysaccharide. This result underpins the challenge of identifying new exopolysaccharide-associated operons and operon classes, owing to their highly mosaic nature and the multiple evolutionary origins of each functionally equivalent subunit found within them, coupled with the lack of a robust technique to accurately identify and functionally annotate them in a high-throughput manner.

Dannheim and colleages have anecdotally proposed that the hypothetical genes we named *ccsZABHI* may be responsible for the secretion of an acetylated glucan by *C*. *difficile* [60]. This work cited the observation of a similar acetylated glucan in *Clostridium acetobutyliticum*, which was noted to possess these same putative genes [60,61]. However, the polymer was proposed to be involved in either exopolysaccharide or capsular biosynthesis. The biosynthesis of capsular polysaccharides in bacteria is carried out by structurally and functionally distinct systems from exopolysaccharide synthases that have been expertly reviewed by others [62]. Here, the structural and functional model we have proposed for *ccsZABHI* (Fig 2) does not support their role in capsule biosynthesis, as none of the predicted structures, functions, or homology of these proteins are in line with the requirements for the described capsular biosynthesis systems. Thus, our analysis suggests that the *ccsZABHI* locus independently identified by Dannheim and colleagues encodes a series of proteins functionally similar to known cellulose exopolysaccharide synthases in Gram-negative bacteria. Furthermore, we have expanded these findings to show that these genes are widely distributed among other pathogenic and non-pathogenic species of class Clostridia.

### The overall structure of CcsZ

We engineered and synthesized the soluble domain of *ccsZ* from *C*. *difficile*, corresponding to residues 30–340 of the GenPept entry WP_077724661.1, into the pET21 expression vector to generate a recombinant His-tagged expression construct of CcsZ. Protein from this construct expressed well, yielding up to 75 mg/L of culture and purified to near homogeneity following nickel affinity chromatography and anion exchange chromatography, making it an excellent candidate for structure determination. Our CcsZ crystals grew in space group *P* 2_1_ and diffracted X-rays well to 1.75 Å resolution with a single copy of CcsZ in the asymmetric unit. We solved the crystal structure of CcsZ using the molecular replacement technique based upon the model of apo-*Tm*Cel5A (PDB ID 3AMD) as a search template. Model building and subsequent refinement resulted in an improved *R*_work_ and *R*_free_ of 0.227 and 0.260, respectively. The model covers almost the entirety of the CcsZ construct, from residues 38 to 337 in the sequence, although residue Ser91 could not be reliably built into the model due to a lack of electron density. The complete data collection and refinement statistics are available in Table 1. The completed structure of CcsZ was deposited to the Protein Data Bank (PDB) under accession code 6UJE.

**Table 1:** Diffraction data processing, refinement statistics and model validation.

	CcsZ apo	CcsZ-BGC
**Data collection**		
Wavelength (Å)	0.9795	0.9795
Space group	P 1 2_1_ 1	P 1 2_1_ 1
Unit cell parameters; Å,°	a = 56.95 b = 45.63 c = 59.94 α = 90, β = 94.99, γ = 90	a = 57.02 b = 46.53 c = 61.09 α = 90, β = 94.50, γ = 90
Resolution, Å	43.04–1.75 (1.813–1.75)	31.69–2.00 (2.071–2.00)
Total no. reflections	60071 (5845)	43557 (4313)
No. of unique reflections	30543 (2983)	25294 (2157)
Redundancy	2.0 (2.0)	2.0 (2.0)
Completeness, %	96.85 (87.20)	99.50 (99.58)
Average *I/*σ(*I*)	9.0 (0.99)	17.97 (2.4)
*R*_merge_, * %	0.08 (3.62)	0.02 (0.28)
CC^1/2^	0.998 (0.229)	1 (0.903)
CC*	1 (0.611)	1 (0.974)
**Refinement**		
*R*_work_/*R*_free_^†^	0.227 / 0.260	0.238 / 0.287
No. of non-H atoms		
Total	2676	2650
Protein	2556	2556
Ligands	1	12
Water	120	82
Average B-factors, ^‡^ Å^2^		
Protein	43.83	44.22
Ligands	30.0	30.0
Water	45.89	42.17
rmsd		
Bond lengths, Å	0.05	0.009
Bond angles,°	1.16	1.15
Ramachandran plot^‡^		
Total favoured, %	94.95	93.94
Total allowed, %	4.71	5.72
PDB accession	6UJE	6UJF

Values in parentheses correspond to the highest resolution shell. *I/σ(I)*: intensity of a group of reflections divided by the standard deviation of those reflections.

*R_merge_ = ∑∑ |*I*(k) − <*I*>|/∑ *I*(k), where *I*(k) and <*I*> represent the diffraction intensity values of the individual measurements and the corresponding mean values. The summation is over all unique measurements.

^†^*R*_work_ = ∑ ||F_obs_|− k|F_calc_||/|F_obs_|, where F_obs_ and F_calc_ are the observed and calculated structure factors, respectively. *R*_*f*ree_ is the sum extended over a subset of reflections excluded from all stages of the refinement.

^‡^As calculated using MolProbity [63].

**Fig 3:**
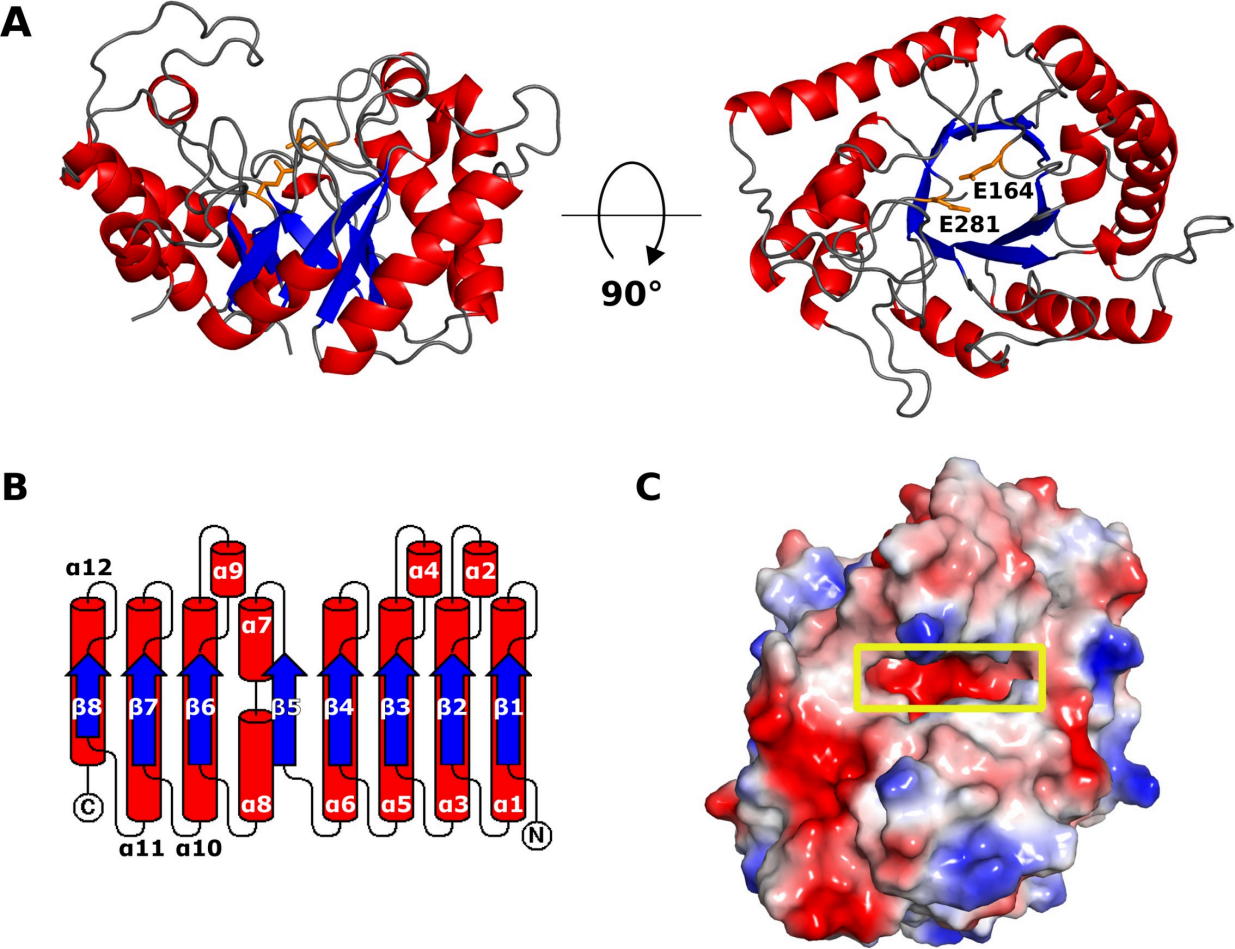
The overall structure of CcsZ. (A) The (α/β)_8_ fold of CcsZ is shown as a cartoon representation from a front (left) and top (right) view. The catalytic resides Glu-164 and Glu-281 are shown in orange. (B) Topology cartoon of the CcsZ fold, coloured as in panel A. (C) The substrate-binding cleft of CcsZ (yellow box) has an overall strong negative charge, an unusual feature for GH-5 enzymes.

In agreement with typical GH-5 enzymes, CcsZ folds into an overall structure adopting a distorted TIM barrel fold, in which an (α/β)_8_ barrel is formed at the core of the fold by eight parallel β-strands (Fig 3A and 3B). This β-barrel motif is flanked by a series of eight partially distorted α-helices packed against the core β-strands, which are connected by extended loops along the C-terminal face of the β-barrel motif. The extended C-terminal loops shape a deep cleft that is typical of GH-5, where both the active site and the cleft for substrate accommodation are located. Interestingly, we observed this groove to be strongly negatively charged in CcsZ, which is not a typical feature of GH-5 (Fig 3C). The GH-5 consensus catalytic acid/base and nucleophile residues are present in CcsZ as Glu-164 and Glu-281, respectively (Fig 3A). Our structure of apo-CcsZ superimposed excellently on apo-*Tm*Cel5A, with a root mean square deviation (r.m.s.d.) of 0.677 Å across 252 equivalent Cα atoms, as expected given the reasonably high sequence identity (43%) between these proteins and the highly conserved GH-5 fold.

### Comparison to known GH-5 structures

Carbohydrate-Active Enzyme family 5 (CAZy GH-5) is an exceptionally large family containing >15,000 known and predicted enzymes. In keeping with the size of GH-5, the substrate specificity of this family is widely variable and encompasses numerous carbohydrate structures, with some members demonstrating strict specificity for single glycan structures, while other members have demonstrated promiscuity with activity on many distinct glycans [64]. The diversity of substrate preference in the GH-5 family is also matched by the wide variety of chemical properties among GH-5 enzymes, such as temperature or pH optima [64–68]. In efforts to delineate the diversity of this large family of glycosyl hydrolases, phylogenetic analyses were performed and numerous sequence-based phylogenetic subfamilies with characteristic substrate preferences were created [64]. Under this classification, Aspeborg and colleagues placed CcsZ into GH-5 subfamily 25 (GH-5_25), a small subfamily of enzymes belonging primarily to thermophilic bacteria, although two other GH-5_25 enzymes from *C*. *difficile* were also classified in GH-5_25 [64]. It was not surprising that, based upon the molecular phylogenetics of GH-5_25, the *Tm*Cel5A enzyme we used as the search model for molecular replacement of CcsZ is also among the most closely related to CcsZ of all GH-5 sequences [64].

**Fig 4:**
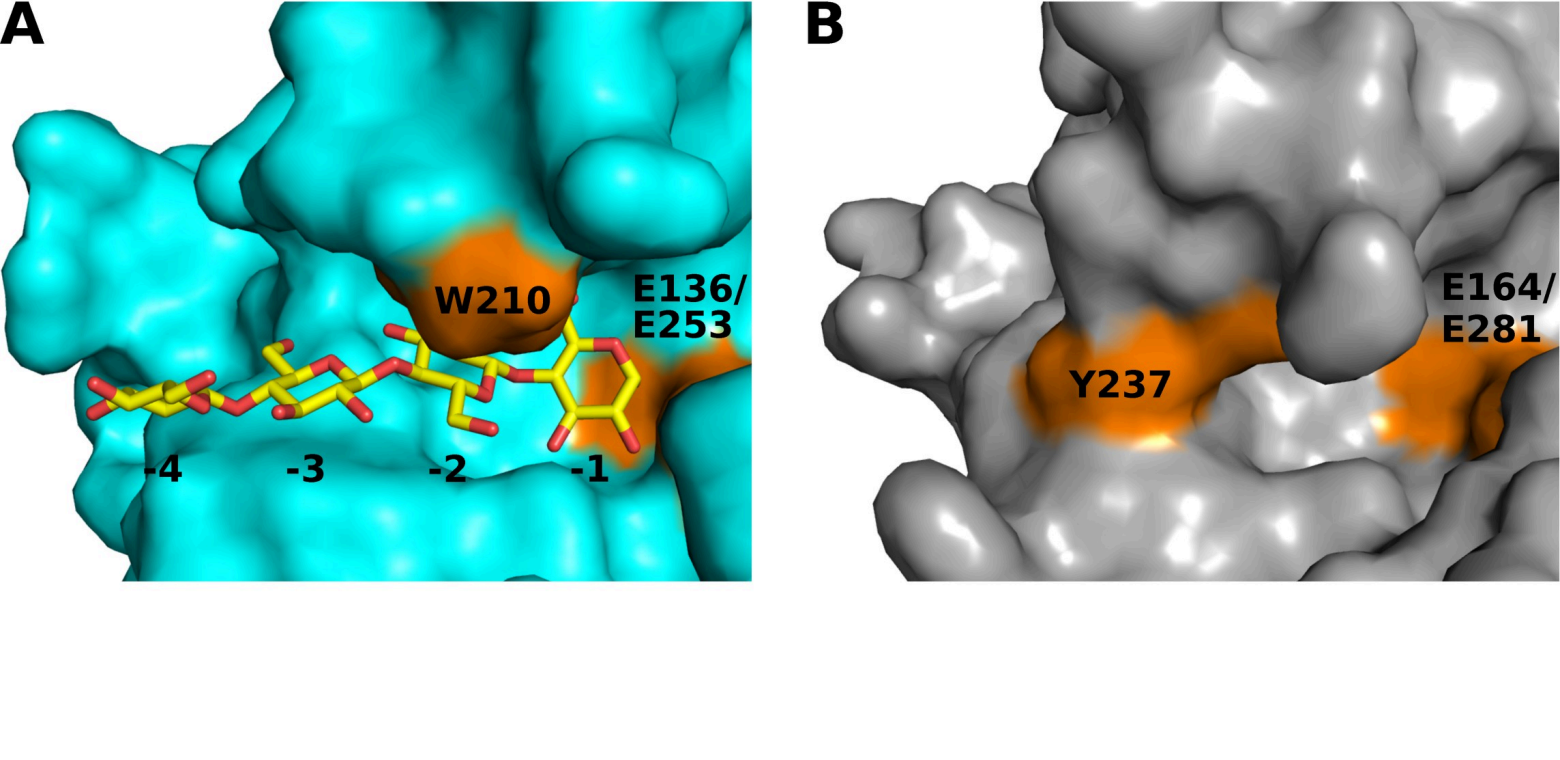
Substrate accommodation by CcsZ and *Tm*Cel5A. (A) The cellotetraose-bound structure of *Tm*Cel5A. The cellotetraose molecule is bound at subsites adjacent to the active site residues Glu-136 and Glu-253, guided by ring-stacking with Trp-210. (B) The equivalent substrate-binding site of CcsZ. The active site residues are present as Glu-164 and Glu-281. The equivalent to the -2 subsite, shaped by W210 in *Tm*Cel5A, is obstructed by the orientation of Tyr-237 in CcsZ, preventing substrate accommodation in the same fashion.

GH-5_25 is reported as a polyspecific subfamily of GH-5 that possesses multiple activities [64]. Notably, *Tm*Cel5A was reported to exhibit activity on both mannan and glucan polymers with β-(1,4) linkage, including linear and branched polysaccharides [69]. Supporting this finding, the crystal structure of apo-*Tm*Cel5A was also reported alongside structures of *Tm*Cel5A ligand complexes, including *Tm*Cel5A bound to cellotetraose, mannotriose, glucose and cellobiose. We compared the structure of *Tm*Cel5A in complex with cellotetraose (PDB ID 3AZT) to CcsZ in an effort to understand how their substrate accommodation might differ. The *Tm*Cel5A-cellotetraose complex also superimposed well over CcsZ, with an r.m.s.d. of 0.620 Å over 242 equivalent Cα atoms. A comparison of the substrate-binding cleft, informed by the positioning of the cellotetraose ligand of *Tm*Cel5A, demonstrated a clear difference between the subsite architectures of the two enzymes (Fig 4). The residues that contribute to the -1 and -2 subsites of *Tm*Cel5A appear to be partially conserved in CcsZ, present as H123, H124, N163, Y225 and N48. However, in *Tm*Cel5A, W210 shapes the face of the -2 subsite and stacks with glucopyranose rings, but in CcsZ, the equivalent Y237 occupies a position that would serve as the -3 subsite in *Tm*Cel5A; thereby resulting in a substrate binding cleft in CcsZ that terminates at the equivalent -2 subsite.

*Tm*Cel5A was also reported to be highly thermostable [68], a feature rationalized by a larger fraction of buried atoms, a smaller accessible surface area and the presence of shorter unstructured loops as compared to the structure of a mesophilic GH-5 cellulase from *Clostridium cellulolyticum* (*Cc*Cel5A; PDB id 1EDG) [65]. However, for the purpose of comparison, it is worth noting that *Cc*Cel5A does not belong to GH-5_25 but instead to GH-5_4, which includes *endo*-β-(1,4) glucanases specific for xyloglucan, as well as licheninases and xylanses. To assess if CcsZ possessed similar features attributed to the thermal stability of *Tm*Cel5A, we uploaded our structure to the PDBePISA server. This analysis revealed both a solvent-accessible surface area for CcsZ of 13497 Å^2^ and a proportion of buried atoms of 0.50 that was most similar to *Tm*Cel5A (13720 Å^2^ and 0.51) rather than *Cc*Cel5A (15,410 Å^2^ and 0.46). Furthermore, CcsZ possessed unstructured loops more similar to *Tm*Cel5A (r.m.s.d. 0.677 Å across 252 Cα atoms) than *Cc*Cel5A (r.m.s.d. 1.053 Å across 172 Cα atoms) based on superimposition of the structures.

After comparison of our structure of CcsZ to other related GH-5 structures, we found the relatively high sequence identity and structural similarity between CcsZ and *Tm*Cel5A, rather than *Cc*Cel5A, (*i*.*e*., 41% vs 18% identity) surprising, based on our expectation that CcsZ would be highly specific for an *O-*acetylated cellulose exopolysaccharide, in contrast to the broad specificity of *Tm*Cel5A for varied mannan and glucan structures. We also did not anticipate CcsZ to possess features associated with extended thermal stability given that *C*. *difficile* is a mesophilic bacterium and that the biofilm phenotype has been reported at temperatures of 25–37°C [8,34]. Accordingly, we set out to biochemically characterize CcsZ to further explore these properties.

### The product-bound structure of CcsZ

Next, in order to experimentally resolve the subsite architecture and mechanism of substrate accommodation by CcsZ, we attempted to solve the structure of CcsZ in complex with cello-oligosaccharides. We were able to grow crystals in a distinct but chemically similar condition to our apo-CcsZ crystals and successfully introduced cellotriose following crystal growth but prior to crystal harvesting. These crystals diffracted X-rays to 1.65 Å resolution and also grew in the space group *P* 2_1_ containing a single polypeptide in the asymmetric unit (Table 1). We solved the structure of our ligand complex with molecular replacement using the structure of the apo-form as the search model. The ligand-bound structure was in complete agreement with the apo-form and although the experimental resolution was higher for the ligand complex crystals than the apo-form, low completeness was observed for the high-resolution data and so the ligand complex structure was refined only against data with a maximum resolution of 2.0 Å. Refinement of the ligand complex structure resulted in an *R*_work_ and *R*_free_ of 0.238 and 0.287, respectively (Table 1). The final model of the CcsZ-ligand complex also covered almost the entirety of the protein, from residues 39–340 and included the missing Ser91 from the apo-form.

**Fig 5:**
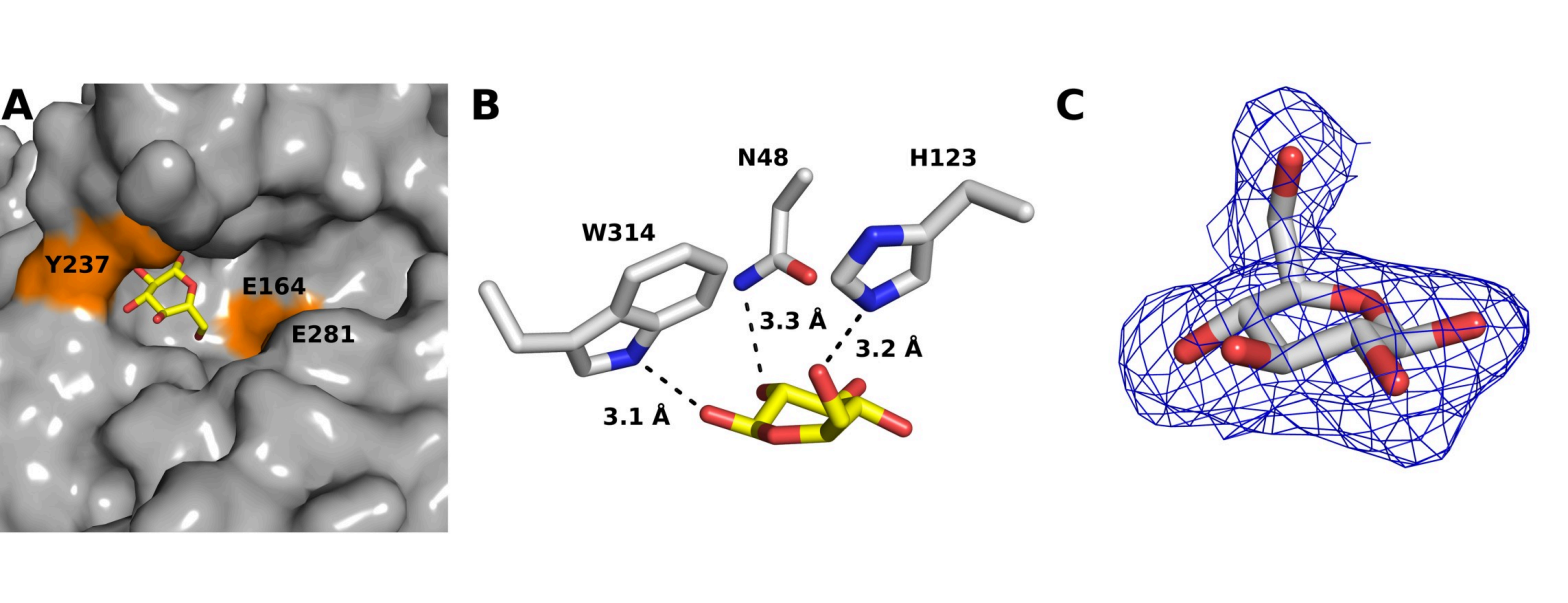
The product-bound structure of CcsZ. (A) A glucose molecule is bound at the apparent -2 subsite adjacent the catalytic architecture and confined by the orientation of Y237. (B) The bound glucose molecule makes polar contacts with W314 via the C1 hydroxyl and N48 via the C2 hydroxyl and H123 via the C6 hydroxyl. (C) The polder omit map electron density (blue mesh) for the glucose ligand contoured at 3 σ.

Following refinement of the complex structure, we observed a Fourier electron density peak, clearly interpretable in both the Fourier 2 *F*_*o*_*—F*_*c*_ electron density map at 1.0 σ contour level and the *F*_*o*_*—F*_*c*_ map at 3.0 σ contour level. This density corresponded well to an apparent glucopyranose ring near the active site. Subsequent ligand fitting placed glucose into this density with a promising real-space correlation coefficient (RSCC) of 0.714 in an unambiguous orientation, with placement aided by the strong electron density observed for the oxygen atom of the C6 hydroxyl. We then calculated a Polder omit map for the glucose ligand, resulting in an improved electron density that allowed refinement of the glucose ligand to a final RSCC of 0.861 (Fig 5C). The bound glucose makes polar contacts with the side-chain N atom of N48 via the C2 hydroxyl, the N atom of the H123 side chain via the C6 hydoxyl and the N atom of the W314 side chain via the C6 hydroxyl, with approximate distances of 3 Å in all cases (Fig 5B). The final refined structure of the CcsZ-glucose complex was deposited to the PDB under accession code 6UJF.

The observation of a single glucose residue bound to CcsZ when a trisaccharide was soaked into the crystals suggested to us that the bound ligand represented an enzymatic product rather than a bound substrate or glucosyl intermediate, which was not surprising given the observed activity of our CcsZ construct specifically on glucan substrates (noted below). Accordingly, this glucose molecule was bound at the equivalent of the -2 subsite in *Tm*Cel5A, but appears spatially confined by the side chain of Y237 and the loop presenting W314, both of which shifted only trivially between our apo- and glucose-bound structures with an all-atom r.m.s.d. of 0.206 Å across 2232 equivalent atoms (Fig 5A). Comparatively, the Gram-negative cellulose hydrolase BcsZ (a GH-8 enzyme), which was resolved both in apo-form (3QXF) and as a complex with cellopentaose (3QXQ), displays a substrate-binding architecture whereby the +1 and +2 subsites are angled at approximately 60° from the -1 to -4 subsites at the nonreducing side of the catalytic center [30]. Thus, structural plasticity of the substrate-binding groove and induced-fit distortion of longer saccharide substrates is certainly possible in these enzymes and is also conceivable in CcsZ, given the structure we observed.

### CcsZ is an *endo*-β-(1,4)-glucanase

**Fig 6:**
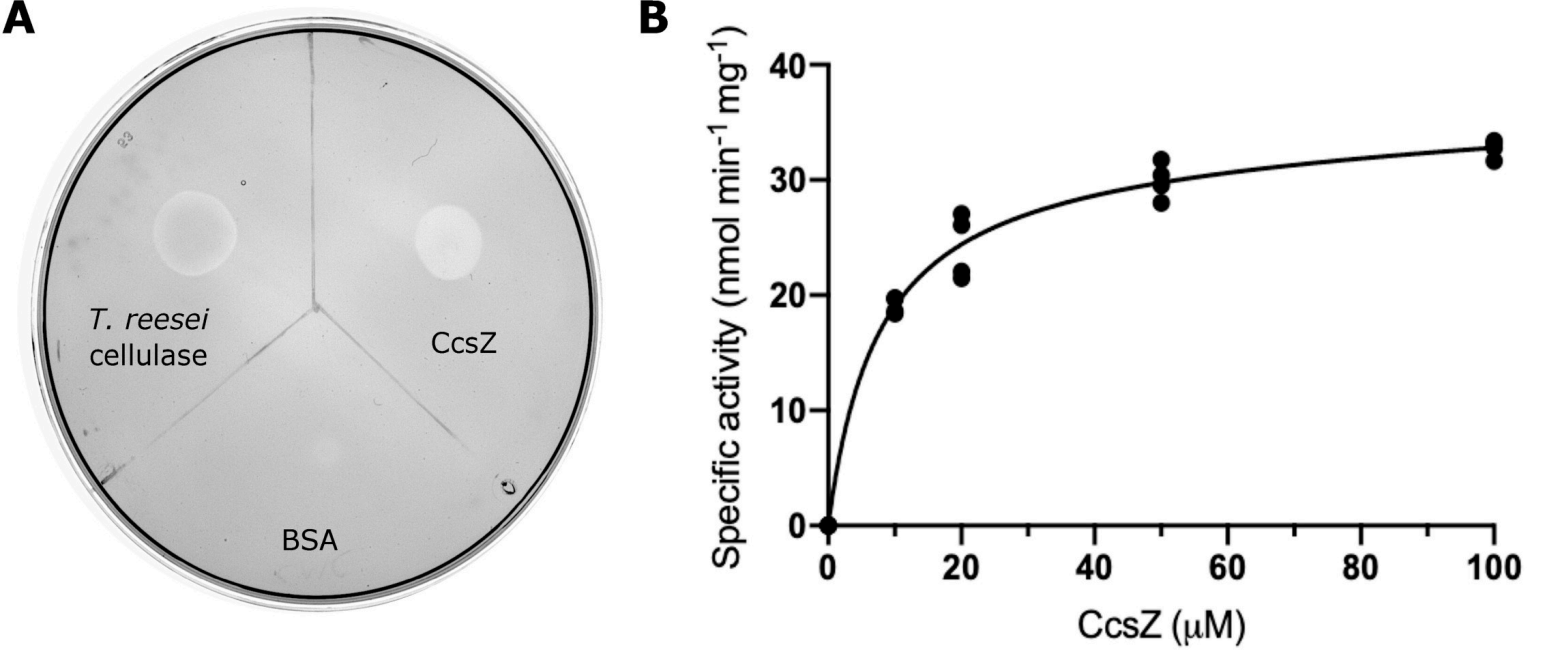
CcsZ is a β-glucanase. (A) CMC hydrolysis by CcsZ and the *T*. *reesei* cellulase cocktail positive control is evident by the zone of clearing in the Congo Red stained CMC plate-based zymogram. (B) CMC hydrolysis by CcsZ. The reducing sugar concentration of a CMC solution (8.3 g/L) was increased by treatment with CcsZ over 4 h at 37°C in a CcsZ concentration-dependent manner.

To test if CcsZ was in fact capable of hydrolytic activity on cellulose, we assessed its activity on the soluble substrate analogue carboyxmethylcellulose (CMC) first using the plate-based zymogram method reported by Mazur and Zimmer for BcsZ [30]. Following staining with Congo Red, a zone of clearing was observed suggesting CcsZ was capable of cleaving the glucosidic bonds of CMC after 1 h incubation (Fig 6A). We also performed this same plate-based zymogram experiment using 0.8% (w/v) concentrations of either CMC, hydroxyethyl cellulose (HEC) and beechwood xylan, or 0.4% (w/v) curdlan over a 24 h incubation. As expected, we observed that CcsZ was active on CMC and HEC but had only limited cleavage of xylan or curdlan (S1 Fig).

**Fig 7:**
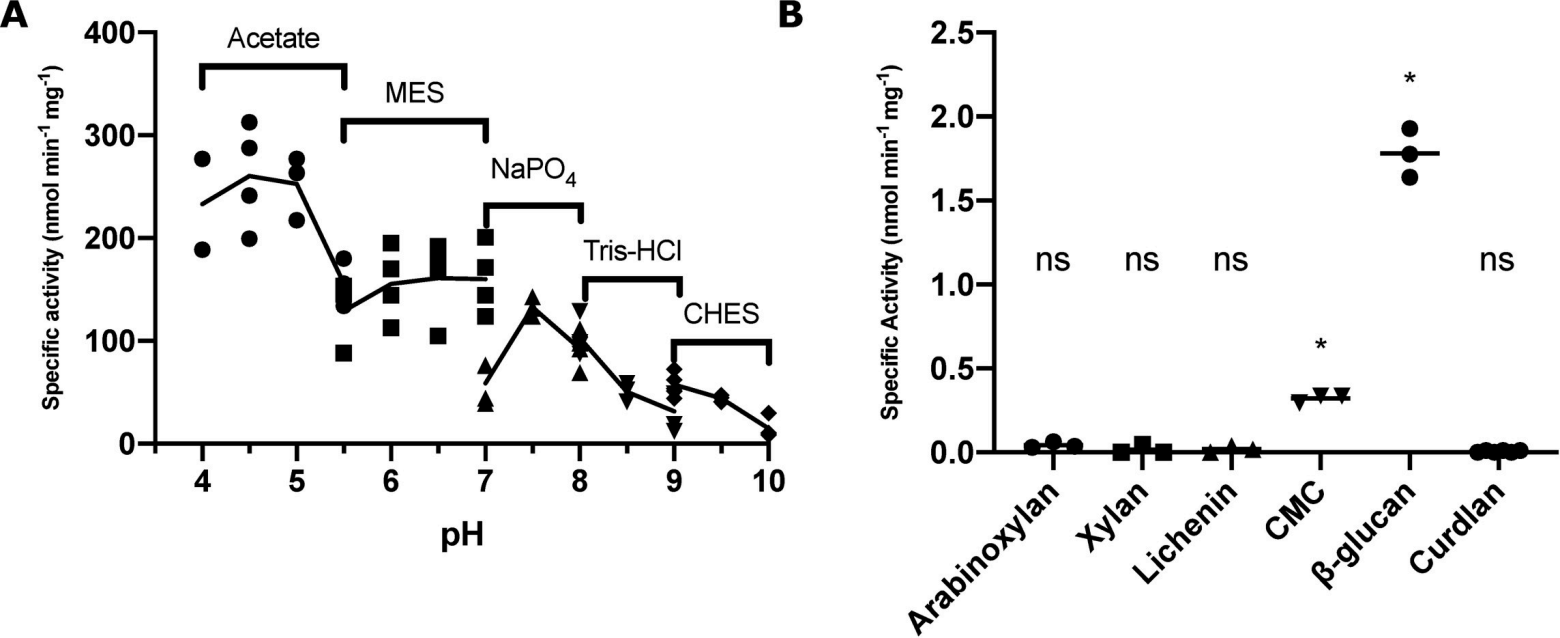
pH and substrate specificity of CcsZ. (A) The pH profile of CcsZ displays a clear preference for acidic conditions with a pH optimum of 4.5. Activity was calculated using the DNS method with solubilized CMC substrate. Buffer solutions used (50 mM) are listed above data points. (B) Substrate utilization profile of CcsZ using common GH-5 substrates. CcsZ exhibited five-fold greater activity on mixed-linkage β-glucan as compared to CMC when assayed using the DNS method. CcsZ activity on arabinoxylan, lichenin, xylan and β(1,3)-linked curdlan was not significantly different from an enzyme-free control under our assay conditions. Assays were performed with at least n ≥ 3 replicates for all groups. ns = not significant and asterisks denote significant activity (t-tests, t(2) = 23.33 and p = 0.0018 for CMC, t(2) = 21.30 and p = 0.0022 for β-glucan). All other groups were not significantly different from 0 (p > 0.05).

Subsequently, we sought to measure CcsZ activity on CMC quantitatively using the dinitrosalicylic acid (DNS) reducing sugar assay, where enzyme activity was calculated from our raw data using a standard curve of glucose prepared using the DNS method. We observed an increase in reducing sugar concentration following incubation with CcsZ in a concentration-dependent manner, further indicating CcsZ is capable of using CMC as a substrate (Fig 6B). We also tested CcsZ with CMC under a range of pH buffer conditions to measure pH stability. We found CcsZ to have a pH optimum of approximately 4.5, which is typical of other characterized GH-5 enzymes (Fig 7A) [65–67]. Although CcsZ was most active under acidic conditions, we found CcsZ was tolerant of a wide variety of pH and buffer conditions and still demonstrated detectable hydrolase activity on CMC across all pH values we tested (pH 4–10). CcsZ activity was reduced two-fold between pH 4.5 and 7.5, with a particular loss in activity under alkaline conditions, showing a six-fold reduction in activity at pH 9.5.

To assess substrate specificity, we also tested the common GH-5 polysaccharide substrates arabinoxylan, xylan, lichenin and β-glucan, along with β-(1,3)-linked curdlan, using the same DNS reducing sugar assay. We did not observe a significant increase in reducing sugar concentration following prolonged CcsZ incubation (*i*.*e*., 24 h) with arabinoxylan, xylan, or lichenin under our conditions (Fig 7B), consistent with the expected activity of CcsZ on cellulose exopolysaccharides and its classification in GH-5_25. However, we observed CcsZ exhibited 5-fold greater activity on β-glucan, a polymer with mixed β-(1,3) and β-(1,4) linkages. In agreement with our structural data, this observation suggested that carboxymethylcellulose was actually a poor CcsZ substrate owing to the stearic constraints of the carboxymethyl groups. Instead, activity on a β-glucan is likely more representative of the minimal, unbranched structure of cellulose exopolysaccharides and others have also reported that β-glucan is a superior substrate mimetic to CMC for GH-5 strict *endo-*β-glucanases [66]. In a follow-up analysis with curdlan, we did not observe a significant increase in reducing sugar concentration with this solely β-(1,3)-linked substrate; thereby indicating that the activity on β-glucan must have been limited to the β-(1,4) linkages, which is consistent with cellulose as the proposed preferred substrate for CcsZ.

**Fig 8:**
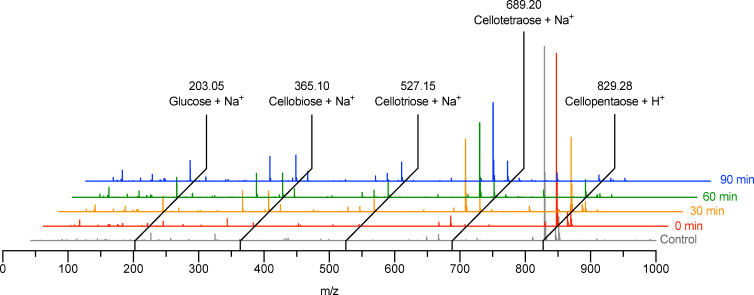
Representative time-resolved mass spectra of the CcsZ products. CcsZ is capable of mixed *exo-* and *endo*-glucanase activity on substrates at least 4 saccharide units in length, since all possible glucanase products were observed with complete digestion of the starting cellopentaose material after 90 min incubation with CcsZ.

*Exo*-acting activities are rare among GH-5 enzymes, with the majority of characterized GH-5 enzymes characterized as *endo-*acting [64], although it is worth noting that this is a challenging biochemical distinction to establish experimentally and that these activities may not be mutually exclusive. In the case of CcsZ, an unusual substrate-accommodation cleft in addition to our structure of CcsZ bound to a glucose monosaccharide suggested that CcsZ may exhibit strict *exo*-glucanase activity, which warranted further investigation. To assess the regioselectivity of CcsZ, we incubated the enzyme with the mock substrate cellopentaose (G_5_) and analyzed the enzymatic products by liquid chromatography-mass spectrometry (LC-MS; Fig 8). Our enzyme-free control contained only the m/z species for the intact G_5_ starting material, indicating the substrate was not solvent-labile. In the mass spectra of the CcsZ enzymatic products, we observed predominantly m/z species that corresponded to sodiated ions for glucose monosaccharide (G_1_), cellobiose disaccharide (G_2_), cellotriose trisaccharide (G_3_) and cellotetraose (G_4_) species. In addition, we also observed in very low abundance the unreacted and intact starting material (G_5_) among the CcsZ products, which were completely digested after 90 min elapsed incubation. This data suggested CcsZ is capable of mixed *exo-* and *endo*-glucanase activity on substrates at least 4 saccharide units in length, at least for the minimal substrate cellopentaose. Although glucose monosaccharides were released, the accumulation of di- and tri-saccharide species after prolonged enzymatic digestion suggests the *exo*-glucanase activity of CcsZ is negligible compared to its *endo-*glucanase activity. This mixed activity also accounted for the existence of a glucose monosaccharide as an enzymatic product, which we were able to structurally resolve bound to CcsZ.

**Fig 9:**
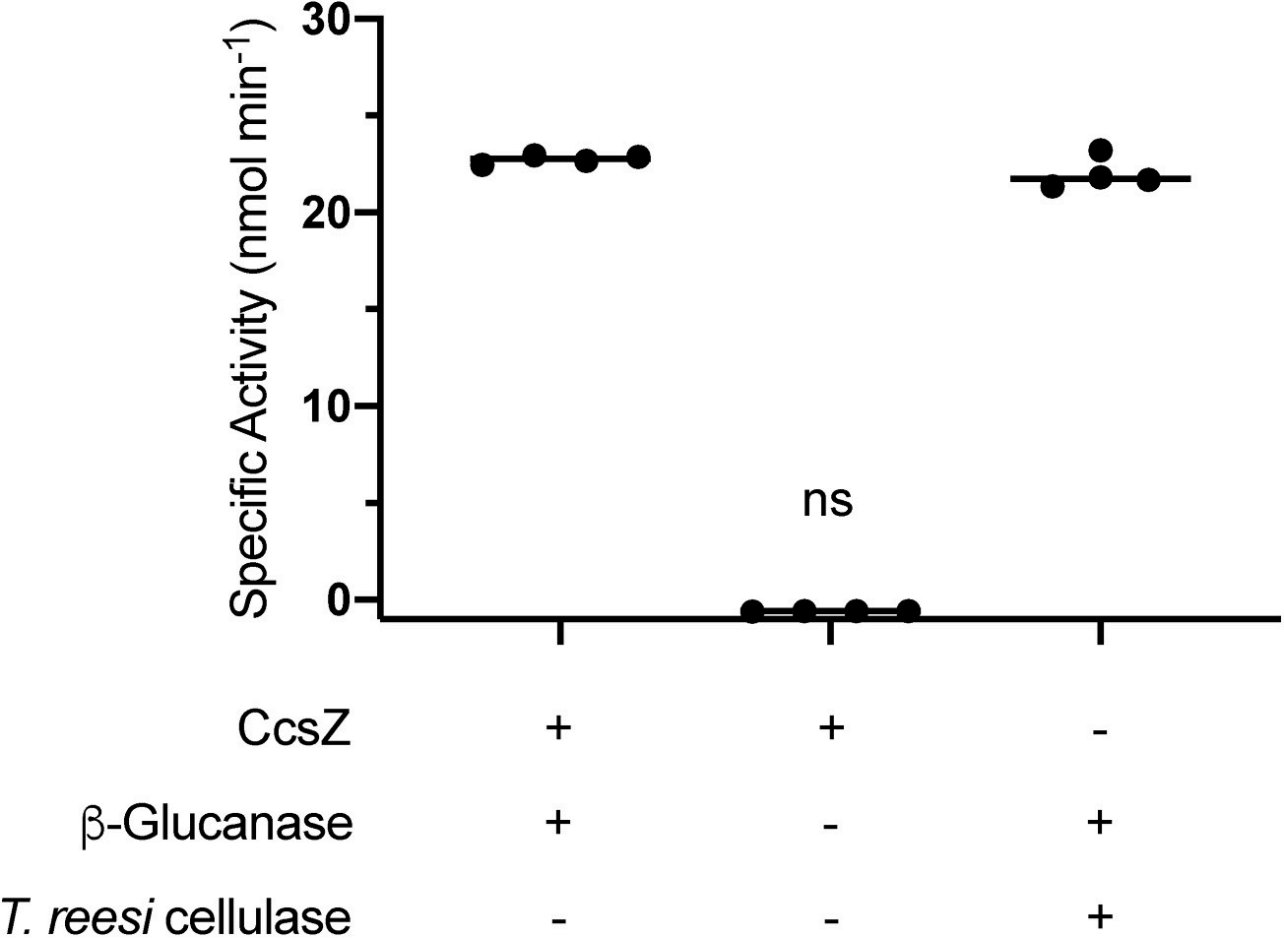
CcsZ is an *endo*-β-glucanase. Endocellulase activity was assessed using a bimodified cellopentaose substrate that contains terminal *p*-nitrophenyl and 3-ketobutylidene groups. CcsZ and the positive control (a cellulase cocktail from *Trichoderma reesei*) demonstrated endocellulase activity in the coupled enzyme assay with an ancillary *exo*-β-glucosidase. CcsZ did not demonstrate *exo*-activity, since degradation of the *endo*-released products and concomitant release of *p*-nitrophenolate was not observed in the absence of the supplied *exo*-β-glucosidase. Data are the results of at least 4 replicates. ns = not significant (ANOVA, F (3, 12) = 3770, q = 1.920, p = 0.1834).

To further asses CcsZ, we analyzed the *endo*- vs *exo*- activity using a K-CELLG5-4V Cellulase Kit (Megazyme, Ireland), which experimentally tests for endocellulase activity using a bimodified cellopentaose substrate that contains terminal *p*-nitrophenyl and 3-ketobutylidene groups. Upon incubation with endocellulase, the modified glucan is cleaved internally; thereby producing exposed terminal glucose subunits that are cleaved by an ancillary β-glucosidase to yield free glucose and *p*-nitrophenolate (*p*NP). The coupled enzyme assay (*i*.*e*., containing the tested endocellulase plus the manufacturer supplied *exo*-β-glucosidase) is terminated and the *p*NP colour is assessed upon addition of Tris stopping reagent. Thus, concentrations of *p*NP are directly proportionate to endocellulase activity as the chromophore cannot be released without the initial *endo*-glucanase activity that cleaves the internal β-(1,4)-linkages. In our analysis of CcsZ, we observed formation of *p*NP that verified CcsZ has endocellulase activity (Fig 9). A parallel reaction without *exo*-β-glucosidase yielded no formation of the colorimetric *p*NP, which suggests that CcsZ possesses no observable *exo*-activity over the time course of the reaction (*i*.*e*., exocellulase activity was only observed in the coupled assay with *exo-*β-glucosidase present). Thus, our results are in keeping with the literature on GH-5 enzymes, of which the majority have *endo*-acting activity and rarely exhibit *exo*-activity [64] and are also consistent with other cellulases found in cellulose synthase clusters that are proposed to release nascent chains from the cell into the biofilm [30,32,70].

## Conclusions

Here, we demonstrate the presence of the *ccsZABHI* locus that bears sequence similarities to known Gram-negative cellulose exopolysaccharide biosynthesis systems and propose for the first time a model of cellulose synthesis in Gram positives (Fig 2). Our subsequent analysis of the cognate glycosyl hydrolase, CcsZ, reveals the Clostridial cellulose synthase contains a functionally analogous, but structurally distinct, glycosyl hydrolase from the known Gram-negative cognate glycosyl hydrolase BcsZ. The findings we present here represent an entry point to an understanding of the molecular mechanisms governing cellulose exopolysaccharide production in the important and well-studied human pathogens *Clostridioides difficile*, *Clostridium botulinum* and *Clostridium perfringens*, as well as other Gram-positive bacteria.

## Supporting information

S1 Fig CcsZ activity assessed using plate-based zymogram analysis. Plates contain 0.8% (w/v) each CMC (A), HEC (B), beechwood xylan (C) and 0.4% (w/v) curdlan (D). CcsZ was capable of complete CMC hydrolysis, resulting in a total loss of Congo Red staining where spotted on the agar, but only partial or no degradation was observed for HEC, xylan and curdlan.
(TIF)

S1 File (PDF)

S2 File (PDF)
