# Supplementary figures and images for: Identification of the Clostridial cellulose synthase and characterization of the cognate glycosyl hydrolase, CcsZ
Source: PLoS One. 2020 Dec 2;15(12):e0242686. doi: 10.1371/journal.pone.0242686 (PMC7710045; doi:10.1371/journal.pone.0242686)

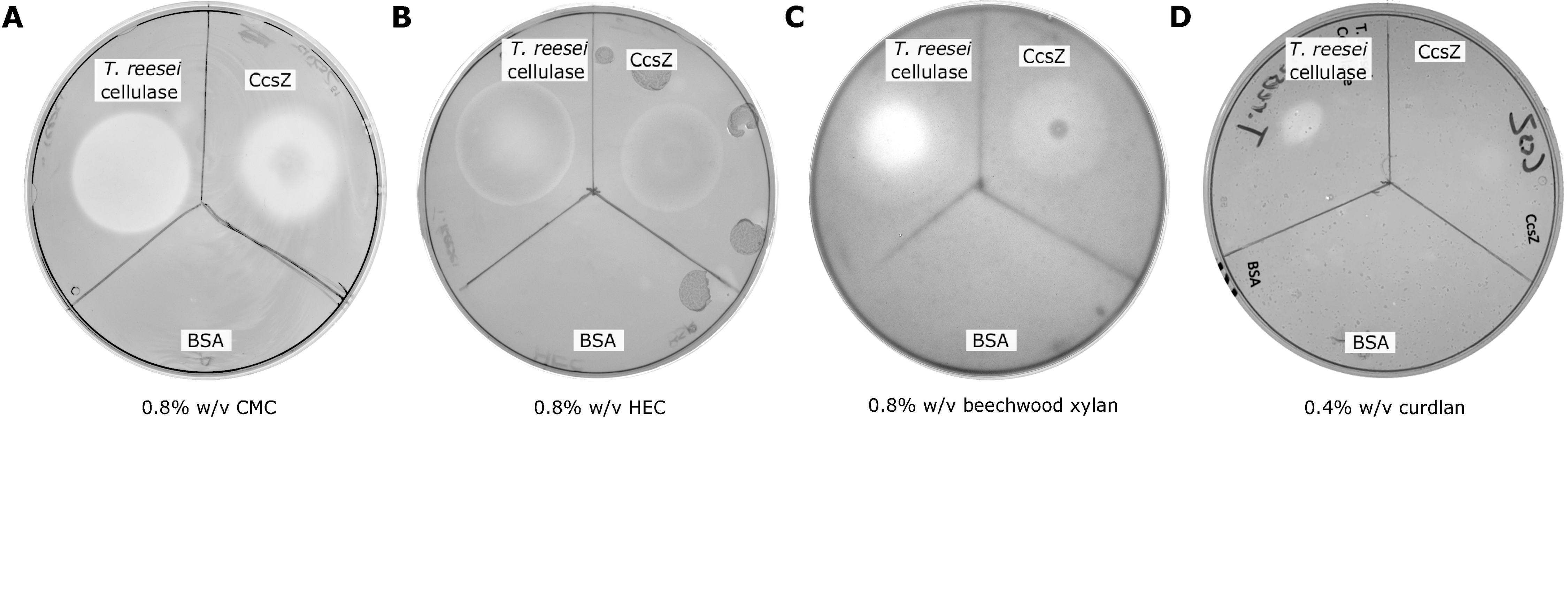

Supplement: S1 Fig — Plates contain 0.8% (w/v) each CMC (A), HEC (B), beechwood xylan (C) and 0.4% (w/v) curdlan (D). CcsZ was capable of complete CMC hydrolysis, resulting in a total loss of Congo Red staining where spotted on the agar, but only partial or no degradation was observed for HEC, xylan and curdlan. (TIF) [file pone.0242686.s001.tif]
